# Upstream deregulation of calcium signaling in Parkinson’s disease

**DOI:** 10.3389/fnmol.2014.00053

**Published:** 2014-06-17

**Authors:** Pilar Rivero-Ríos, Patricia Gómez-Suaga, Elena Fdez, Sabine Hilfiker

**Affiliations:** Instituto de Parasitología y Biomedicina “López-Neyra,” Consejo Superior de Investigaciones CientíficasGranada, Spain

**Keywords:** Parkinson’s disease, dopamine, calcium, mitochondria, endoplasmic reticulum, lysosomes, Golgi

## Abstract

Parkinson’s disease (PD) is a major health problem affecting millions of people worldwide. Recent studies provide compelling evidence that altered Ca^2^^+^ homeostasis may underlie disease pathomechanism and be an inherent feature of all vulnerable neurons. The downstream effects of altered Ca^2^^+^ handling in the distinct subcellular organelles for proper cellular function are beginning to be elucidated. Here, we summarize the evidence that vulnerable neurons may be exposed to homeostatic Ca^2^^+^ stress which may determine their selective vulnerability, and suggest how abnormal Ca^2^^+^ handling in the distinct intracellular compartments may compromise neuronal health in the context of aging, environmental, and genetic stress. Gaining a better understanding of the varied effects of Ca^2^^+^ dyshomeostasis may allow novel combinatorial therapeutic strategies to slow PD progression.

## INTRODUCTION – WHICH NEURONS DIE IN PD?

Parkinson’s disease (PD) is an incurable late-onset neurodegenerative disorder which is strongly associated with aging, as evidenced by the exponential increase in incidence above the age of 65 (de Rijk et al., 1997; de Lau et al., 2004). Due to extended life expectancy, the prevalence of PD is estimated to double by 2030. Therefore, deciphering the molecular mechanisms underlying the disease, with the aim of developing novel disease-modifying therapies, has become an urgent and crucial task in PD-related research. Whilst PD is a disease of neurons, not all neurons are affected. The motor symptoms of PD, such as resting tremor, bradykinesia, and rigidity are clearly linked to the death of dopamine (DA) neurons in the substantia nigra pars compacta (SNc). Similarly, the clinical gold-standard treatment of L-DOPA (3,4-dihydroxy-L-phenylalanine), a DA precursor, indicates that DA neurons are crucial to the disease. However, the neuropathological hallmarks of PD, which are the presence of proteinaceous intracellular deposits called Lewy bodies or Lewy neurites in surviving neurons, are more distributed and not exclusive to DA neurons. Non-DA neurons which show pathology in PD include cholinergic neurons in the dorsal motor nucleus of the vagus (DMV) and basal forebrain (BF), noradrenergic neurons in the locus ceruleus (LC), and serotonergic neurons in the raphe nuclei (RN; Braak et al., 2004). Neurodegeneration is also not evident in all dopaminergic neuronal populations. For example, DA neurons in the ventral tegmental area (VTA) are relatively unaffected (Matzuk and Saper, 1985; Kish et al., 1988; Ito et al., 1992; Damier et al., 1999). Thus, elucidating why the diverse neurons are at risk for degeneration is essential if we want to formulate testable hypotheses as to the cause(s) underlying PD.

## WHY DO NEURONS DIE IN PD – FROM DOPAMINE TO MITOCHONDRIA

Distinct mechanisms have been proposed to account for the preferential loss of DA neurons in PD. One hypothesis proposed that DA itself may be the culprit, as oxidation of cytosolic DA and its metabolites can lead to the production of cytotoxic free radicals and oxidative stress (Greenamyre and Hastings, 2004). However, since not all dopaminergic neurons are at risk in PD, and since elevating DA levels in PD patients by L-DOPA administration does not accelerate the progression of PD (Fahn, 2005), DA unlikely is the principal culprit, even though its effects may further worsen the cellular deficits related to oxidant stress and/or protein aggregation triggered by other means (see below).

Another hypothesis has linked PD to mitochondrial dysfunction (Henchcliffe and Beal, 2008; Schapira, 2008; Vila et al., 2008). Mitochondria are crucial organelles for cellular energy production. The transport of electrons down the electron transport chain (ETC) releases energy which is used by complex I, III, and IV to pump protons from the mitochondrial matrix to the mitochondrial intermembrane space, creating a proton gradient and an electrochemical gradient across the mitochondrial inner membrane, the latter of which is being used by ATP synthase to convert ADP to ATP. Mitochondria comprise one of the major cellular producers of reactive oxygen species (ROS), as electrons in the ETC are occasionally captured by oxygen to produce superoxide anion radicals, with complex I and III being the major culprits for production of these radicals (Cali et al., 2011).

There is extensive evidence for mitochondrial involvement in both sporadic and genetic PD. Toxins such as MPTP, rotenone, and paraquat, which inhibit complex I, can cause a Parkinsonian phenotype (Betarbet et al., 2000; Przedborski et al., 2004). In addition, postmortem tissue samples derived from the SNc from sporadic PD patients display a drastic decrease in the activity of complex I (Mann et al., 1994). A deficit in ETC can cause mitochondria-derived oxidative stress in the form of ROS and other radicals. Indeed, the decreased activity of complex I in PD patients seems due to oxidative damage (Keeney et al., 2006) and also affects other cellular components such as lipids and DNA (Zhang et al., 1999). Oxidative damage may also be responsible for the high levels of somatic mitochondrial DNA (mtDNA) deletions in SNc DA neurons (Bender et al., 2006; Kraytsberg et al., 2006), and the physical proximity of mtDNA to the site of ROS generation may indeed make them a vulnerable target. Since seven proteins involved in the formation of complex I are encoded by the mitochondrial genome, this may give rise to further ETC dysfunction and oxidative stress, leading to accelerated loss of SNc DA neurons.

However, the observed decrease in complex I deficiency in homogenates from nigral tissue from PD patients is too big to be restricted to SNc DA neurons, and only a proportion of PD patients show complex I inhibition in the SNc (Jenner, 2001). In addition, whilst toxins such as the herbicide rotenone cause ubiquitous complex I inhibition, dopaminergic degeneration is observed in the SNc, but not in the VTA area (Betarbet et al., 2000). Thus, inhibition of mitochondrial complex I activity *per se* cannot explain the selective vulnerability of neurons which die in PD.

## WHY DO NEURONS DIE IN PD – PACEMAKING, Ca^2+^ DYSHOMEOSTASIS, AND OXIDANT STRESS

**FIGURE 1:**
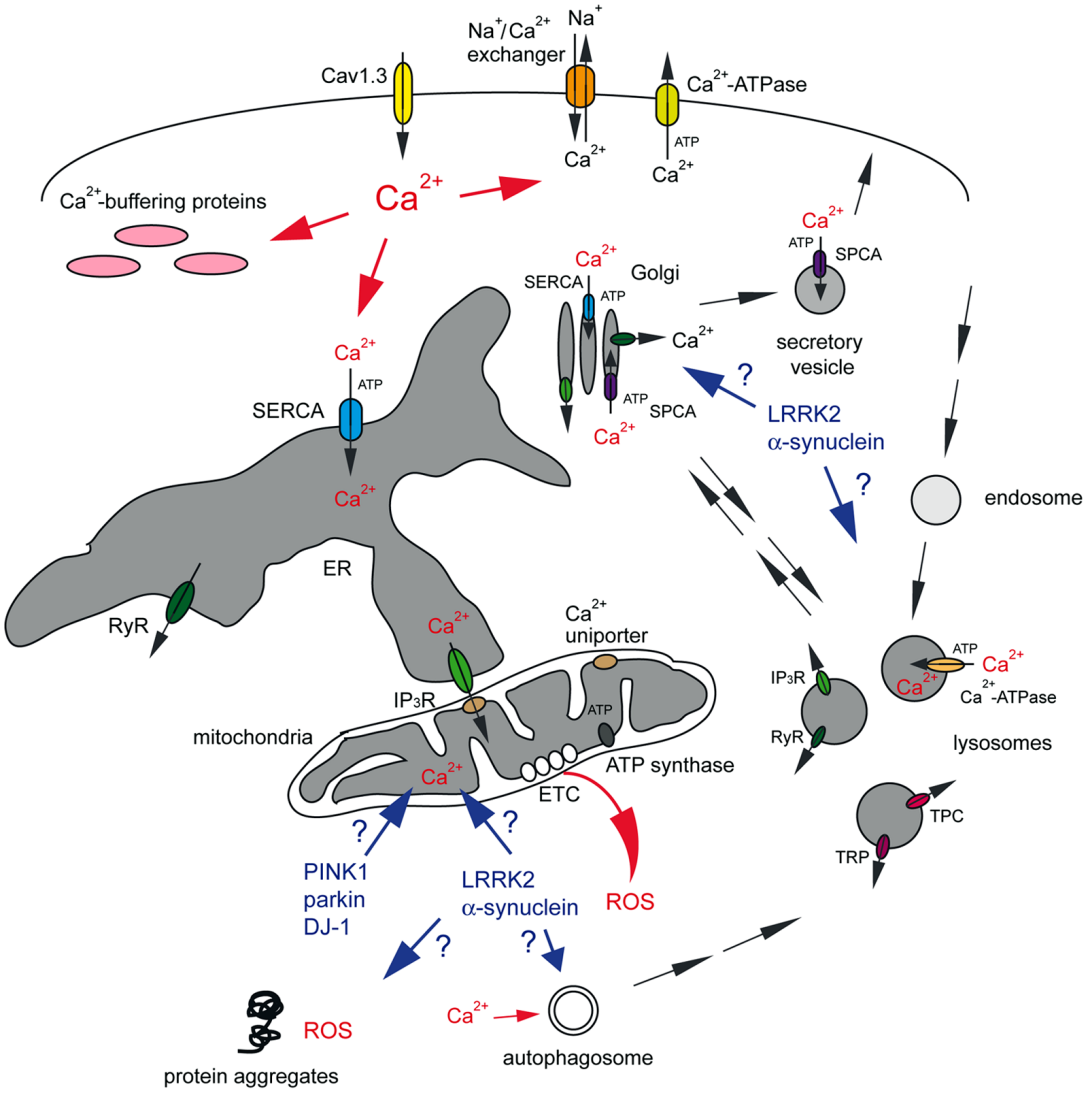
**Abnormal Ca^2+^ signaling in SNc DA neurons may cause mitochondrial oxidant stress, proteostasis deficits and eventual cell death**. Vulnerable neuronal populations display spontaneous slow pacemaking activity employing Cav1.3 L-type Ca^2^^+^ channels, prominent Ca^2^^+^ currents and low intrinsic Ca^2^^+^ buffering capacities. Ca^2^^+^ inside the neuron can be transported back across the plasma membrane either via plasma membrane Ca^2^^+^-ATPase at the cost of ATP consumption, or through the Na^+^/Ca^2^^+^ exchanger which uses the Na^+^ gradient across the plasma membrane. Ca^2^^+^ is rapidly sequestered by interactions with Ca^2^^+^ buffering proteins or taken up into a variety of intracellular organelles. The ER uses a high-affinity Ca^2^^+^-ATPase [the sarco-endoplasmic reticulum Ca^2^^+^-ATPase (SERCA)] to pump Ca^2^^+^ into the ER lumen at the cost of ATP consumption. This pump is also present on cis and medial Golgi membranes, whilst secretory vesicles employ a secretory pathway Ca^2^^+^-ATPase (SPCA) which is also be present on the trans Golgi complex. Ca^2^^+^ uptake into acidic organelles is mediated by a molecularly unidentified Ca^2^^+^-ATPase. Ca^2^^+^ flows back into the cytosol from the ER lumen through IP_3_ receptors (IP_3_R) or ryanodine receptors (RyR). IP_3_R are also present on cis and medial Golgi membranes, RyR on trans Golgi membranes, and RyR, TRP and TPC channels are present on acidic organelles. Mitochondria, often in close apposition to the ER or plasma membrane, can take up Ca^2^^+^ into the matrix through a mitochondrial Ca^2^^+^ uniporter. Ca^2^^+^ transfer between ER and mitochondria involves the IP_3_R on the ER membrane. Ca^2^^+^ within mitochondria is necessary for proper ETC function to generate ATP by ATP synthase, but mitochondrial Ca^2^^+^ overload can cause mitochondrial oxidant stress (ROS). Toxins as well as familial mutations in PINK1, parkin and DJ-1 affect mitochondrial ATP production and Ca^2^^+^ handling, even though the molecular details remain to be determined. The effects of familial mutations in LRRK2 and α-synuclein on mitochondrial functioning are even less clear, but those mutant proteins may cause additional deficits in proteostasis through mechanisms involving Ca^2^^+^-regulated events such as autophagy. This may also include alterations in the trafficking of Golgi-derived vesicles to the plasma membrane, resulting in changes in vesicle secretion and in the steady-state levels of surface receptors. Golgi deficits may cause altered trafficking of enzymes destined to lysosomes, with concomitant deficits in lysosomal degradative capacity, or alterations in retromer-mediated retrieval from endolysosomes back to the Golgi. Finally, changes in acidic store Ca^2^^+^ levels may affect various endo-lysosomal trafficking steps or the degradative capacity of acidic organelles *per se*. For further details see text.

A hypothesis, put forward by Surmeier’s group, suggests that specific and shared physiological features are responsible for the risk of a subset of neurons to degenerate in PD (Guzman et al., 2010; Surmeier et al., 2011; Goldberg et al., 2012), and comprises probably the best working model to explain disease pathomechanism to date (**Figure 1**).

Neurons are electrically excitable, using steep electrochemical gradients (mainly Na^+^ and K^+^ gradients) across their plasma membrane to integrate incoming chemical signals, and pass them on to other neurons. Voltage-dependent Ca^2^^+^ channels in most neurons are only opened by strong depolarization during an action potential. These channels close relatively slowly during membrane repolarization, such that the total Ca^2^^+^ influx during a spike is very sensitive to spike duration. To minimize global increases in Ca^2^^+^, neurons which need to spike at high frequencies tend to restrict Ca^2^^+^ entry by keeping spikes very brief, and tend to express Ca^2^^+^ buffering proteins to help manage intracellular Ca^2^^+^ levels (Augustine et al., 2003).

In contrast to many other neurons, SNc DA neurons are autonomously active in the absence of synaptic input (Grace and Bunney, 1983). Such pacemaking activity is necessary to maintain a basal DA tone in the striatum; without it, movement ceases (Surmeier and Schumacker, 2013). Whilst most neurons rely on Na^+^ to drive this pacemaking activity, SNc DA neurons also engage L-type Ca^2^^+^ channels with a Cav1.3 pore-forming subunit (Bonci et al., 1998; Puopolo et al., 2007). Although not strictly necessary for pacemaking, L-type Ca^2^^+^ channels help support pacemaking (Guzman et al., 2009). SNc DA neurons exhibit slow, broad spikes, causing a significant increase in intracellular Ca^2^^+^ levels, and they lack relevant intrinsic Ca^2^^+^ buffering capacity (Foehring et al., 2009; Guzman et al., 2009). The combination of these features, namely spontaneous activity that can be intrinsically generated, broad action potentials, prominent Ca^2^^+^ currents and low intrinsic Ca^2^^+^ buffering capacities are common to all neurons at risk for neurodegeneration in PD, irrespective of their neurotransmitter content (Surmeier and Schumacker, 2013). In contrast, relatively non-affected VTA DA neurons, whilst also slow pacemaking neurons, have low L-type Ca^2^^+^ channel densities and express high levels of the Ca^2^^+^ buffering protein calbindin (German et al., 1992; Khaliq and Bean, 2010).

## GETTING RID OF Ca^2+^ – AN ENERGETICALLY COSTLY PROCESS

The shared physiological phenotype of at-risk neurons means that they will have a larger burden to handle increased intracellular Ca^2^^+^ levels. As Ca^2^^+^ is a universal second messenger, controlling a wide variety of cellular events ranging from regulation of enzyme activity to programmed cell death, it is under tight homeostatic control (Petersen et al., 2005). Pumping Ca^2^^+^ out of the cytosol is an energy-consuming process. Cytosolic Ca^2^^+^ levels are set to around 100 nM, which is 20,000-fold lower than the Ca^2^^+^ concentration in the extracellular space. This contrasts with the concentration differences of Na^+^ and K^+^ ions across the plasma membrane, which is in the range of 10–30-fold. Thus, thermodynamic considerations dictate that it will be energetically much more expensive to move Ca^2^^+^ ions across the plasma membrane as compared to Na^+^ or K^+^ ions (Surmeier and Schumacker, 2013).

Ca^2+^ ions are removed from the cytosol by either exchangers or pumps. Exchangers, such as the Na^+^/Ca^2^^+^ exchanger use the Na^+^ gradient to move Ca^2^^+^ ions out of the cytosol. Pumps, such as the plasma membrane Ca^2^^+^-ATPase, use ATP to drive the movement of ions against a concentration gradient. Ca^2^^+^ buffering proteins further help to decrease the free Ca^2^^+^ concentration. Importantly, Ca^2^^+^ which is not rapidly pumped out of the neuron is sequestered into intracellular organelles including the endoplasmic reticulum (ER), mitochondria, Golgi, and lysosomes (**Figure 1**; Berridge et al., 2000; Rizzuto, 2001; Pinton et al., 2008; Lloyd-Evans and Platt, 2011; Kaufman and Malhotra, 2014).

How the increased demand for Ca^2^^+^ handling causes increased risk for degeneration of the vulnerable neuronal populations remains to be fully elucidated. One hypothesis proposes that due to their high basal ATP consumption rates related to Ca^2^^+^ handling, vulnerable neurons will have a lesser bioenergetic or respiratory reserve, which is defined as the difference between the maximum capacity for ATP generation by oxidative phosphorylation and the basal ATP consumption rate (Nicholls, 2008). A smaller respiratory reserve may put these neurons at risk when their metabolic demands increase, such as during bursts of spiking or upon toxin exposure. Indeed, when ATP levels are not sufficient to meet demands, a deterioration of the membrane potential would be followed by massive Ca^2^^+^ influx and cell death.

The increased metabolic demand of SNc neurons may also give rise to an increase in the basal level of mitochondrial oxidant stress, as high rates of metabolic activity cause increased ROS production (**Figure 1**; Lee et al., 2001). In support of this, pacemaking in SNc neurons was shown to generate mitochondrial oxidant stress, which was not apparent in neighboring VTA DA neurons (Guzman et al., 2010). Such oxidant stress was largely prevented in the presence of L-type Ca^2^^+^ channel antagonists, clearly implicating those channels and the resultant increase in intracellular Ca^2^^+^ as culprits for downstream oxidant stress generated by high demands for mitochondrial ATP production.

Mitochondrial oxidant stress causes mild mitochondrial depolarization or uncoupling (Guzman et al., 2010), which leads to a decline in energy production and generation of ROS, causing damage to proteins, lipid, and DNA. In accordance with this, mtDNA deletions are significantly greater in SNc DA neurons from older as compared to younger subjects, and from neurons from PD patients as compared to unaffected individuals (Bender et al., 2006; Kraytsberg et al., 2006), with no changes observed in other brain areas. The accumulation of mtDNA deletions, with effects on mitochondrial respiratory chain function, will thus lead to further bioenergetic deficiency that manifests over time.

## GETTING RID OF Ca^2+^ – NOT JUST A PROBLEM OF ENERGY

It is clear that cytosolic Ca^2^^+^ levels have to be maintained within a small range of concentrations for optimal survival of SNc DA neurons (Michel et al., 2013). However, apart from the extra bioenergetic burden to control intracellular Ca^2^^+^ levels, altered Ca^2^^+^ handling by various intracellular organelles may threaten neuronal viability as well. Indeed, mitochondrial oxidant stress in SNc DA neurons can be diminished when limiting mitochondrial Ca^2^^+^ uptake, without affecting pacemaking (Guzman et al., 2010). This is important, as it suggests that mitochondrial oxidant stress may be the consequence of increased mitochondrial Ca^2^^+^ load, rather than a mere reflection of the need for increased ATP production.

Ca^2^^+^ is well-known to modulate mitochondrial function. The Ca^2^^+^ uniporter uses the mitochondrial membrane potential to take Ca^2^^+^ up into the mitochondrial matrix (Kirichok et al., 2004; Santo-Domingo and Demaurex, 2010), where it increases ATP production by stimulating enzymes of the tricarboxylic acid (TCA) cycle, and thus helps to maintain increased metabolic demands associated with electrical activity and influx of Ca^2^^+^ (McCormack and Denton, 1990). However, too much Ca^2^^+^ in mitochondria compromises mitochondrial function by causing a transient collapse of the mitochondrial membrane potential (McCormack and Denton, 1990), which thus transiently halts the production of ATP.

The mitochondrial Ca^2^^+^ uniporter drives rapid and massive Ca^2^^+^ entry at high cytosolic Ca^2^^+^ concentrations only thought to be reached in microdomains near plasma membrane Ca^2^^+^ channels and Ca^2^^+^ release channels on the ER. Indeed, the primary intracellular organelle dealing with Ca^2^^+^ homeostasis is thought to be the ER (Berridge, 2002; Verkhratsky, 2005). The ER is responsible for the coordinated production, delivery, and degradation of proteins in a process called proteostasis. It forms a continuous intracellular network which extends throughout the somatodendritic tree (Choi et al., 2006), and contains high-affinity ATP-dependent transporters [(sarco-ER Ca^2^^+^-ATPase (SERCA)] to move Ca^2^^+^ from the cytoplasm into the ER lumen. Ca^2^^+^ sequestered in the ER can be released at sites where it can be pumped back across the plasma membrane, or can be used locally to modulate cellular function (Verkhratsky, 2005). The Ca^2^^+^ store in the ER is highly interconnected with other intracellular Ca^2^^+^ stores, such that ER Ca^2^^+^ dyshomeostasis will affect Ca^2^^+^ handling in other organelles as well. For example, inositol 1,4,5-trisphosphate (IP_3_) receptors which reside at direct ER-mitochondrial contacts termed MAMs (mitochondria-associated ER membranes) allow for direct flux of Ca^2^^+^ from ER into mitochondria (Csordas et al., 2006; Rizzuto and Pozzan, 2006; Kaufman and Malhotra, 2014), which may then lead to the mitochondrial Ca^2^^+^ overload described above (**Figure 1**). Indeed, stimulation of Ca^2^^+^ release from the ER by ryanodine, accompanied by an increase in cytosolic Ca^2^^+^ levels, was found to protect DA neurons from spontaneous or induced neurodegeneration (Guerreiro et al., 2008). Thus, relieving the Ca^2^^+^ load in the ER, without significantly causing Ca^2^^+^ transfer from ER to mitochondria through IP_3_ receptors, may prove beneficial to the survival of DA neurons, possibly via preventing ER-mediated mitochondrial Ca^2^^+^ overload. Altered ER Ca^2^^+^ concentrations are also associated with altered changes in cytosolic Ca^2^^+^ concentration upon ER release, and thus can affect the downstream signaling functions of this organelle (Morikawa et al., 2000; LaFerla, 2002).

Apart from its signaling function, Ca^2^^+^ plays an inherently important role for the functioning of the ER by acting as an allosteric regulator of protein processing and folding. Depletion of ER Ca^2^^+^ stores induces ER stress and the unfolded protein response (Paschen and Mengesdorf, 2005). Too much intraluminal ER Ca^2^^+^ may compromise proteostasis as well. For example, L-type Ca^2^^+^ channel blockers have been shown to restore folding and lysosomal delivery of mutant lysosomal enzymes responsible for a variety of lysosomal storage diseases (Mu et al., 2008). Similarly, decreasing ER Ca^2^^+^ levels by SERCA inhibitors seems to enhance the folding and plasma membrane trafficking of mutant cystic fibrosis transmembrane conductance regulator (CFTR; Egan et al., 2002, 2004). Precise Ca^2^^+^ imaging experiments will be required to determine the intraluminal ER Ca^2^^+^ levels upon such treatments. Nevertheless, these data indicate that altering ER Ca^2^^+^ homeostasis can have profound effects on folding and trafficking of proteins destined to other subcellular locations including lysosomes and the plasma membrane (**Figure 1**), with obvious downstream effects both on plasma membrane functioning/signaling and lysosomal degradative capacity.

## INTRACELLULAR Ca^2+^ STORES AND Ca^2+^ HANDLING: THE NEGLECTED PLAYERS

In addition to the ER and mitochondria, two other compartments deserve attention as significant intracellular Ca^2^^+^ store. The first is the Golgi apparatus, which shares some functions and biochemical markers with the ER. The Golgi complex is a highly dynamic intracellular organelle which processes and sorts membrane proteins derived from the ER to the cell surface, secretory vesicles or lysosomes, and which also receives retrograde transport input. Thus, damage to neuronal Golgi structure can have important functional consequences for protein and vesicular trafficking (Fan et al., 2008). Interestingly, Golgi fragmentation has been observed in nigral neurons from PD patients (Fujita et al., 2006), and recent studies indicate that increased neuronal activity causes reversible Golgi fragmentation in a manner dependent on Ca^2^^+^-calmodulin-dependent protein kinase (Thayer et al., 2013). It will be interesting to determine whether Golgi fragmentation is a shared phenotype of vulnerable neurons in PD, and if it can be modulated by L-type Ca^2^^+^ channel antagonists. In addition, it remains to be seen whether neuronal activity-dependent Golgi fragmentation causes Golgi-derived Ca^2^^+^ release which may alter the spatio-temporal complexity of cellular Ca^2^^+^ signaling.

The Golgi complex serves as a bona fide Ca^2^^+^ store, containing Ca^2^^+^-ATPases, Ca^2^^+^ release channels and Ca^2^^+^-binding proteins (**Figure 1**; Scherer et al., 1996; Pinton et al., 1998; Lin et al., 1999). The Golgi seems to handle Ca^2^^+^ differently dependent on its sub-compartments. Whilst *cis*- and medial Golgi compartments contain the SERCA ATPase and IP_3_ receptors, the trans Golgi takes Ca^2^^+^ up exclusively via SPCA1 (secretory pathway Ca^2^^+^-ATPase isoform 1), and at least in some cells contains ryanodine receptors (Lissandron et al., 2010). Thus, the Golgi can serve as a Ca^2^^+^ store responding to local Ca^2^^+^-induced Ca^2^^+^ release or to second messengers such as cyclic ADP ribose (cADPR) and nicotinic acid adenine dinucleotide phosphate (NAADP) which have been shown to activate ryanodine receptors (Fliegert et al., 2007). Decreasing Ca^2^^+^ in the trans-Golgi complex alters the structure of the entire Golgi apparatus, with effects on sorting of proteins to the plasma membrane through the secretory pathway (Lissandron et al., 2010; Micaroni, 2012). For example, depletion of SPCA1 has been shown to disrupt polarized trafficking, impairing neuronal differentiation, and the generation of functional neurites (Sepúlveda et al., 2009). The mechanism by which intraluminal Ca^2^^+^ in the Golgi may regulate sorting is starting to emerge. For example, sorting of some secretory proteins has been shown to require actin remodeling by ADF/cofilin, SPCA1, and a soluble Golgi-resident Ca^2^^+^-binding protein (von Blume et al., 2011, 2012). Sorting may depend on a transient influx of Ca^2^^+^ into the trans Golgi induced by the binding of ADF/cofilin to SPCA1, which may facilitate the association of secretory proteins with the Golgi-resident Ca^2^^+^-binding protein, acting as a soluble receptor to segregate a subset of secretory proteins (Kienzle and von Blume, 2014). In sum, alterations in intraluminal Ca^2^^+^ concentrations can impact both on cellular Ca^2^^+^ signaling as well as on Golgi structure and secreted protein cargo sorting (Micaroni, 2012), and it will be interesting to determine whether this may cause cell-autonomous deficits for example by altering the formation and trafficking of small dense-core DA-containing vesicles (Bauerfeind et al., 1995), or non-cell-autonomous events such as altering the secretion of neurotrophic factors with downstream effects on dopaminergic cell survival (Kordower and Bjorklund, 2013).

Ca^2^^+^ is also stored in a variety of acidic organelles (Patel and Docampo, 2010). Acidic organelles containing Ca^2^^+^ include endosomes, lysosomes, lysosome-related organelles and secretory granules. Amongst acidic organelles, lysosomes probably comprise the most prominent Ca^2^^+^ stores, and may contain an average free Ca^2^^+^ concentration in the range of 500 μM, similar to the Ca^2^^+^ concentration within the ER (Lloyd-Evans et al., 2008). Ca^2^^+^ uptake into lysosomes is thought to be mediated by pumps. Indeed, purified lysosomes from neutrophils, fibroblasts, and rat liver have been shown to take up Ca^2^^+^ in an ATP-dependent manner (Klempner, 1985; Lemons and Thoene, 1991; Ezaki et al., 1992; Adachi et al., 1996). The molecular nature of the lysosomal Ca^2^^+^-ATPase remains to be determined, even though some data indicate that it may be driven by SERCA3 (López et al., 2005). Alternatively, Ca^2^^+^ loading into lysosomes has been suggested to involve ER Ca^2^^+^ leak, such that small fluctuations in ER Ca^2^^+^ levels may cause large effects on lysosomal Ca^2^^+^ load (Bezprozvanny, 2012). Acidic stores also possess Ca^2^^+^-permeable channels such as IP_3_/ryanodine receptors, TRP channels (transient receptor potential channel superfamily), and TPCs (two-pore channels), which are members of the TRP channel superfamily as well (**Figure 1**). TPC channels located on endosomes and lysosomes have been reported to be targets for NAADP, the most potent Ca^2^^+^ mobilizing messenger (Churchill et al., 2002; Guse and Lee, 2008). However, they do not directly bind to NAADP (Lin-Moshier et al., 2012; Walseth et al., 2012), and their gating properties and ion selectivity have recently been questioned (Wang et al., 2012; Cang et al., 2013). This may be due to the fact that they can heterodimerize in-between themselves as well as with a subset of TRP channels, which are gated by NAADP as well (Patel and Docampo, 2010), and further work will be necessary to elucidate how second messengers such as NAADP may trigger Ca^2^^+^ release from acidic organelles, and the precise channels involved.

Lysosomal impairments seem intricately linked to PD pathogenesis. Lysosomes are the primary degradative organelle in all cell types, and their function is particularly important in non-dividing cells such as neurons. Several diseases associated with lysosomal dysfunction (lysosomal storage diseases) have been identified, and many of them affect brain function. Conversely, many neurodegenerative diseases also exhibit lysosomal dysfunction (Schultz et al., 2011). Lysosomal impairments are observed in sporadic PD brain and toxic as well as genetic rodent models of PD-related neurodegeneration (Dehay et al., 2013). The mechanisms involved may be varied, including defects in the lysosomal delivery of enzymes required for degradation, defects in lysosomal acidification or altered intralysosomal Ca^2^^+^ handling. Importantly, the lysosomal degradative system is characterized by many vesicular fusion events along the endocytic pathway which depend on intraluminal Ca^2^^+^, and lysosomal Ca^2^^+^ is also required for luminal content condensation (Pryor et al., 2000; Luzio et al., 2007). Whilst precise Ca^2^^+^ imaging experiments will be required to determine whether SNc neurons display alterations in intralysosomal Ca^2^^+^ levels, such lysosomal Ca^2^^+^ dyshomeostasis is expected to cause impaired turnover of dysfunctional mitochondria, which would further aggravate mitochondria-derived oxidant stress in vulnerable neurons.

In the context of proteostasis, it is also worthy considering effects of altered intracellular Ca^2^^+^ levels on autophagy, a process employed by cells to get rid of protein aggregates and defunct organelles, and deficits of which are also clearly implicated in PD (Lynch-Day et al., 2012). There is some controversy as to whether increases in Ca^2^^+^ promote or inhibit autophagy. This may be due to the subcellular localization of the source of the Ca^2^^+^ signal and may also depend on cellular state (Decuypere et al., 2011). Under normal conditions, the IP_3_ receptor-mediated Ca^2^^+^ transfer from the ER to mitochondria, which maintains mitochondrial ATP production, seems to inhibit autophagy. In contrast, an increase in cytosolic Ca^2^^+^ concentrations can stimulate autophagy (**Figure 1**; Decuypere et al., 2011). In both cases, this may involve the activity of AMPK, which is activated when cellular ATP levels drop and/or when cytosolic Ca^2^^+^ levels increase. Activation of autophagy, combined with a decrease in lysosomal degradative capacity, may then lead to the observed accumulation of autolysosomal structures observed in PD brains (Anglade et al., 1997).

## PD, AGING, RISK FACTORS, AND GENETICS

Age is clearly the single strongest risk factor for PD. The physiological properties of SNc DA neurons indicate that they will be at a higher risk of age-related cell death due to their enhanced burden of Ca^2^^+^ handling. Indeed, these neurons seem to be lost at a higher rate (5–10% every 10 years) than many other neurons in the brain, some of which do not display significant loss over 60–70 years (Stark and Pakkenberg, 2004). Environmental factors may further alter intracellular Ca^2^^+^ handling, or may impact upon downstream cellular events triggered by Ca^2^^+^ dyshomeostasis, playing either protective or damaging roles. As mentioned above, for example toxins known to cause PD increase mitochondrial oxidant stress, thus impacting upon the same pathway already affected in vulnerable neurons.

Similarly, genetic forms of PD would be expected to converge on pathways affected by altered intracellular Ca^2^^+^ handling. Familial mutations in a variety of genes, with either autosomal-recessive (parkin, PINK1, DJ-1) or autosomal-dominant [(α-synuclein, leucine-rich repeat kinase (2LRRK2)] inheritance account for approximately 10% of PD cases (Trinh and Farrer, 2013). Of those, parkin, PINK1, and DJ-1 are clearly implicated in mitochondrial homeostasis and Ca^2^^+^ handling (Scarffe et al., 2014). For example, DJ-1 seems to protect against mitochondrial oxidant stress evoked by pacemaking in dopaminergic neurons by interfering with mitochondrial uncoupling in response to calcium-induced stress (Guzman et al., 2010). Depletion of DJ-1 seems to decrease expression of certain mitochondrial uncoupling proteins, even though the underlying mechanism(s) remain to be determined. PINK1 has been proposed to contribute to maintaining bioenergetic function of mitochondria by regulating Ca^2^^+^ efflux via the Na^+^/Ca^2^^+^ exchanger, and PINK1 deficiency was reported to cause mitochondrial Ca^2^^+^ overload, resulting in mitochondrial oxidant stress (Gandhi et al., 2009). Other studies indicate that PINK1 deficiency is associated with mitochondrial fragmentation, decreased membrane potential and decreased agonist-stimulated Ca^2^^+^ entry, thus pinpointing to a role for PINK1 in mitochondrial Ca^2^^+^ uptake rather than Ca^2^^+^ extrusion, and concomitant decreased ATP production (Heeman et al., 2011). Similarly, parkin deficiency has been reported to cause mitochondrial fragmentation and ER-mitochondria Ca^2^^+^ crosstalk, thus affecting cellular bioenergetics (Cali et al., 2013). Both parkin and PINK1 cooperate to regulate mitochondrial quality control events such as fission and fusion, degradation of defunct mitochondria by autophagy (mitophagy), mitochondrial transport, and biogenesis (Scarffe et al., 2014). Whilst the molecular mechanism(s) at present remain sketchy, these three proteins seem to be implicated in the same Ca^2^^+^-mediated pathway which is already compromised in sporadic PD (**Figure 1**).

Other proteins implicated in familial PD such as α-synuclein and LRRK2 have been consistently shown to cause dysfunction of the autophagy/lysosomal degradation system (**Figure 1**; Manzoni and Lewis, 2013), but how they may impact upon ER-mitochondrial Ca^2^^+^ handling and mitochondrial oxidant stress is less clear. Autosomal-dominant mutations in LRRK2 have been shown to cause deficits in Ca^2^^+^ homeostasis, leading to mitochondrial depolarization and enhanced mitophagy, which can be prevented by L-type Ca^2^^+^ channel inhibitors (Papkovskaia et al., 2012; Cherra et al., 2013). Greater levels of mtDNA damage can be observed in LRRK2 mutant patient cells as compared to healthy subjects (Sanders et al., 2014), but whether this is due to altered mitochondrial Ca^2^^+^ handling remains to be determined.

Apart from directly affecting mitochondrial Ca^2^^+^ handling, gene products involved in familial PD may also affect Ca^2^^+^ homeostasis in other intracellular organelles such as ER, Golgi, or lysosomes, with downstream effects on proteostasis and protein aggregation. Precise Ca^2^^+^ imaging experiments in the context of both sporadic and familial PD models will be required to reveal possible alterations in intracellular Ca^2^^+^ handling by these distinct organelles. For example, altered lysosomal Ca^2^^+^ levels may be responsible for the observed changes in lysosomal morphology, clustering, and degradative capacity described for mutant LRRK2-expressing cells (MacLeod et al., 2006; Tong et al., 2010; Dodson et al., 2012; Gómez-Suaga et al., 2012; Orenstein et al., 2013). Such changes, concomitant with an increase in cytosolic Ca^2^^+^ levels (Gómez-Suaga et al., 2012), may lead to aberrations in autophagic clearance, followed by a deficit in proteostasis. Impaired proteostasis in the presence of mutant α-synuclein has recently been shown to indirectly increase mitochondrial oxidant stress, suggesting that proteostatic extra-mitochondrial stress may be additive with mitochondrial oxidant stress observed in SNc DA neurons (**Figure 1**; Dryanovski et al., 2013). Whilst the mechanism(s) by which this occurs requires further investigation, it seems to involve NADPH oxidase activity. These data indicate that extramitochondrial oxidant stress may significantly contribute to PD, such that reverting proteostasis deficits may also be therapeutically beneficial in slowing down PD progression. In this context, Golgi-derived proteostasis effects may be worth considering as well, and may underlie altered risk for sporadic (Beilina et al., 2014) as well as familial PD, where Golgi phenotypes have been observed upon mutant α-synuclein and LRRK2 expression (Lin et al., 2009), even though whether this is related to altered Ca^2^^+^ handling in the Golgi remains to be determined. In sum, Ca^2^^+^ dyshomeostasis seems to be central towards our understanding of both sporadic and familial PD, and can affect a plethora of cellular events related to mitochondrial bioenergetics and oxidant stress as well as proteostasis (at the level of the ER, Golgi, and lysosomes) which may in turn increase extramitochondrial-derived oxidant stress to further threaten the viability of affected neurons.

## NOVEL HOPES FOR TREATMENT OPTIONS?

The above-mentioned findings indicate that L-type Ca^2^^+^ channel antagonists may be viable therapeutic targets in the early stages of PD. There are oral antagonists [dihydropyridines (DHP)] available, with good blood–brain barrier permeability and a long record of safe use in humans. Adult SNc DA neurons can compensate for L-type Ca^2^^+^ channel antagonism and continue pacemaking (Chan et al., 2007), and mice do not show obvious motor, learning, or cognitive deficits when treated with L-type Ca^2^^+^ channel antagonists (Bonci et al., 1998), suggesting that these compounds do not alter the functional activity of SNc DA neurons. Indeed, several studies in humans indicate that these compounds diminish the risk of developing PD (Becker et al., 2008; Ritz et al., 2010; Pasternak et al., 2012). However, they do not seem to slow progression of PD (Marras et al., 2012), maybe because of their relatively poor potency against Cav1.3 L-type Ca^2^^+^ channels, or because other factors may become more prominent during disease manifestation. Such factors may in part derive from alterations in intracellular Ca^2^^+^ stores, with the resultant varied downstream effects on cellular proteostasis.

Much work remains to be done before gaining a clearer understanding of the role of Ca^2^^+^ dysregulation in the pathogenesis of PD. It is becoming increasingly clear that abnormal Ca^2^^+^ handling may have pleiotropic effects on a variety of intracellular events resulting in mitochondrial oxidant stress, deficits in ER proteostasis, endolysosomal/autophagic trafficking and alterations in Golgi function which require further investigation. Thus, whilst L-type Ca^2^^+^ channel antagonists may attack the source of the problem, improving the deteriorated cellular functions of mitochondria, ER, lysosomes, or Golgi may be an efficient complementary strategy to attack the varied downstream effects of the increased burden of handling intracellular Ca^2^^+^ in vulnerable neurons. Maybe a feasible future therapeutic strategy should not involve a “hit-hard” principle employed for example to treat cancer patients, but rather a “hit-softly, continue hitting, and hit at multiple places at a time” principle aimed at correcting a combination of cellular deficits derived from improper Ca^2^^+^ handling employing combination-type therapies.

## Conflict of Interest Statement

The authors declare that the research was conducted in the absence of any commercial or financial relationships that could be construed as a potential conflict of interest.
